# Immunisation With Immunodominant Linear B Cell Epitopes Vaccine of Manganese Transport Protein C Confers Protection against *Staphylococcus aureus* Infection

**DOI:** 10.1371/journal.pone.0149638

**Published:** 2016-02-19

**Authors:** Hui-Jie Yang, Jin-Yong Zhang, Chao Wei, Liu-Yang Yang, Qian-Fei Zuo, Yuan Zhuang, You-Jun Feng, Swaminath Srinivas, Hao Zeng, Quan-Ming Zou

**Affiliations:** 1 National Engineering Research Centre for Immunological Products, Department of Microbiology and Biochemical Pharmacy, College of Pharmacy, Third Military Medical University, Chongqing 400038, PR China; 2 School of Basic Medical Sciences, Zhejiang University, Hangzhou, Zhejiang 310058, PR China; 3 Department of Biochemistry, University of Illinois, Urbana, IL, 61801, United States of America; Instituto Butantan, BRAZIL

## Abstract

Vaccination strategies for *Staphylococcus aureus*, particularly methicillin-resistant *S*. *aureus* (MRSA) infections have attracted much research attention. Recent efforts have been made to select manganese transport protein C, or manganese binding surface lipoprotein C (MntC), which is a metal ion associated with pathogen nutrition uptake, as potential candidates for an *S*. *aureus* vaccine. Although protective humoral immune responses to MntC are well-characterised, much less is known about detailed MntC-specific B cell epitope mapping and particularly epitope vaccines, which are less-time consuming and more convenient. In this study, we generated a recombinant protein rMntC which induced strong antibody response when used for immunisation with CFA/IFA adjuvant. On the basis of the results, linear B cell epitopes within MntC were finely mapped using a series of overlapping synthetic peptides. Further studies indicate that MntC113-136, MntC209-232, and MntC263-286 might be the original linear B-cell immune dominant epitope of MntC, furthermore, three-dimensional (3-d) crystal structure results indicate that the three immunodominant epitopes were displayed on the surface of the MntC antigen. On the basis of immunodominant MntC113-136, MntC209-232, and MntC263-286 peptides, the epitope vaccine for *S*. *aureus* induces a high antibody level which is biased to TH2 and provides effective immune protection and strong opsonophagocytic killing activity *in vitro* against MRSA infection. In summary, the study provides strong proof of the optimisation of MRSA B cell epitope vaccine designs and their use, which was based on the MntC antigen in the development of an MRSA vaccine.

## Introduction

*Staphylococcus aureus* (*S*. *aureus*) is an opportunistic bacterial pathogen responsible for a diverse range of human infection diseases [1] [2], including minor skin infections and life-threatening diseases, such as bacteraemia, pneumonia, endocarditis, osteomyelitis, sepsis, and wound infections [3] [4] [5]. These diseases are associated with a high rate of morbidity and mortality, imposing an increasingly high burden on health care resources [6]. In particular, infections of MRSA (methicillin-resistant *S*. *aureus*) that are resistant to vancomycin or multi-antibiotic strategies are now endemic in many health care institutions and communities [7] [8]This requires the exploration of new therapeutic strategies such as an effective vaccine [9].

Manganese is an important metal ion for many pathogens [10] and the uptake of manganese by *S*. *aureus* is achieved by the manganese transport protein complex [11], which is mainly a manganese binding surface lipoprotein (MntC) [12] [13]. MntC is essentially a metal-binding protein, which has been shown to confer protective immunity in animal model systems of *S*. *aureus* infections [4] [14] [15]. In addition, anti-MntC monoclonal antibodies have been identified as binding to *S*. *aureus* cells [16], MntC might be a potential therapeutic target for the development of antibiotics, and MntC could define potential antigen combinations for multi-component vaccines [17].

Antibody response (immune protective) was reported as a major specific immunity resource against MRSA infection [18]. In this study, we found that immunised purified MntC protein is responsible for eliciting anti-MntC IgG immune responses as an immunotherapeutic agent and that it effectively increased immune protection rates against MRSA in a BALB/c systemic infection mouse model, which probably functioned through the B cell immunodominant epitopes of MntC. However, the particular detailed epitope-mapping and protective mechanism of the potential humoral immune response of MntC antigen remain unclear, further the realisation of an epitope-vaccine in MRSA infection remains a challenge.

To elaborate further the humoral immune response of MntC antibody and characterise detailed linear B cell antibody epitopes, the overlapping synthetic peptides were used to detect the MntC-specific antibodies in immunised rMntC vaccinations with mice serum and MRSA-infected post rMntC immunised mice serum, respectively. The linear B-cell epitopes of MntC were completely mapped, and the vaccine basis of immunodominant epitopes of MntC was evaluated. The conservation of all three immunodominant epitopes was then confirmed and located in a 3-d structural model of MntC. Furthermore, we evaluated the efficacy of the immune protection conferred by the immunodominant-epitope vaccine of MntC by using survival rates, antibody response, and opsonophagocytic activity of immunodominant-epitope peptides-specific antibody *in vitro*. Our findings authenticated MntC113-136, MntC209-232, and MntC263-286 as three immunodominant epitopes on the MntC of MRSA and confirmed that the vaccine with three epitope-peptides presented better protective efficacy in mice. Moreover, opsonophagocytic assays indicated that the epitope-vaccine specific IgG was able to kill the *S*. *aureus* bacteria *in vitro*. These studies of MntC epitope will be helpful for understanding the humoral immunity response and epitope-vaccine will be alternative and promising in developing an MRSA vaccine.

## Materials and Methods

### Ethics statement

All animal care and use protocols in this study were performed in accordance with the Regulations for the Administration of Affairs Concerning Experimental Animals approved by the State Council of People's Republic of China. All animal experiments were approved by the Animal Ethical and Experimental Committee of the Third Military Medical University (Chongqing; permit number 2011–04). All surgery was performed under sodium pentobarbital anaesthesia, and animals were sacrificed at the time points indicated below using CO_2_ inhalation: every effort was made to minimise suffering.

### Acquiring recombinant MntC protein

*S*. *aureus* standard strain MRSA252, as described elsewhere [19], was purchased from the American Type Culture Collection (Manassas, VA, USA). The SAR0641 gene encoding the mature protein of *S*. *aureus* MntC (amino acid 25–309) was amplified from the genome of *S*. *aureus* MRSA252 by polymerase chain reaction (PCR) using primers 5′- CTGGGATCCAGCAGTGATAAGTCAAATGGCAAAC-3′ and 5′-ATGCGGCCGCTTATTATTTCATGCTTCCGTG-3′. The PCR product was cloned into an expression vector derived from the pGEX-6p-2 plasmid and expressed in the *Escherichia coli* BL21 (DE3) strain. Isopropyl-b-D-1-thiogalactopyranoside (IPTG) was then added to induce the expression of recombinant protein at 16°C overnight, and rMntC was expressed as a GST fusion protein that facilitated the subsequent purification process.

GST-tagged rMntC proteins were harvested from cleared lysates with glutathione-Sepharose. Next, the recombinant MntC proteins were purified using CaptoTM MMC. The protein eluate was subjected to an endotoxin removal by Triton X-114 phase separation as described previously [20]. Finally, the resulting protein was analyzed by gel-filtration using Superdex^TM^ 200 10/300GL.Purity of Protein was determined using SDS-PAGE and further analyzed using HPLC with a C3 column. The concentration of the resulting protein was determined using the BCA method. The endotoxin content after removal was detected using the tachyplens ameboyto lysate assay.

### Immunisation with rMntC and peptides and challenge infection

To confirm the survival rates of rMntC immune protective as a vaccine against *S*. *aureus*, six to eight week old female BALB/c mice were injected intramuscularly thrice with 40 μg rMntC protein plus an identical volume of complete Freund’s adjuvant (CFA) 25μl at one subcutaneous injection and two booster subcutaneous injections in same volume with incomplete Freund’s adjuvant (IFA) 25μl, or adjuvant alone, on days 0, 14, and 21. Then the immunised BALB/c mice were infected intravenously with MRSA252 (1 × 10^9^ CFU) on day 35, the lethal dose of which was calculated from previous experiments and the survival rates were monitored 14 days after challenge.

To determine and compare the immune protective efficacy of vaccine with CFA/IFA as adjuvant of epitope peptides and rMntC antigen, after the immunodominant epitopes of MntC were identified, we immunised mice with 100 μg KLH-conjugated immunodominant epitope-peptides and 100 μg the mixture Polypeptides, which was prepared with equal distribution of all three immunodominant epitopes (33.3μg/peptide-KLH) with identical CFA/IFA adjuvant and rMntC protein respectively, the procedure of immunisation and infection intravenous with MRSA252 (1 × 10^9^ CFU) was as described above.

To collect MntC-specific serum for identifying immunodominant epitopes, the mice were immunised thrice by rMntC vaccine and intravenously with MRSA252 (5 × 10^8^ CFU infection dose), then sera of immunised rMntC vaccinations were acquired at 7 days after last immunisation and sera in MRSA-infected mice that were administered rMntC vaccinations were acquired at 14 days after the MRSA252 challenge.

In detail, all animals in the survival study were sacrificed by CO_2_ asphyxiation when they became moribund as defined by a combination of ruffled fur, hunched back, and dulled response to stimuli, such as finger probing, when we monitored and recorded the condition of the mice at 0800, 1600, and 2400/0000 daily. Upon completion of all experiments, survivors were sacrificed by CO_2_ overdose in accordance with IACUC policy.

### Peptide synthesis and KLH conjugation

According to the sequence of MntC, 46 synthetic overlapping peptides were constructed from the entire length of MntC of MRSA252 without the headmost 25 amino acid signal peptides. Another irrelevant GST141-158 peptide was synthesised as a negative control; these peptides comprised 18 consecutive amino acid residues, with each 12 amino acids overlapped. To eliminate the defaults of short peptide fragments and improve vaccinated efficacy of animals, peptides were conjugated with Keyhole limpet hemocyanin(KLH). In the conjugation process, the dissolved peptides in Phosphate Buffered Saline (PBS) and glutaraldehyde reacted 30 minutes then conjugated with KLH by dropping persisting 4 hours in room temperature. This synthesis and KLH conjugation of peptides were accomplished by the same company of Chinapeptides, and the purity of all of the aforementioned peptides was expected to be 90%, or higher.

### Epitope mapping with synthetic overlapping peptides

These peptides were dissolved in dimethyl sulfoxide (DMSO) at 1 mg/mL then diluted in hydrogen bicarbonate buffer (pH 9.6) to 5 mM to coat microtitre plates, serum samples from rMntC-immunised mice or infected mice with immunised before infection were diluted 1:300 with antibody diluent. Non-specific binding was prevented by 1% bovine serum albumin (BSA) to block the coated microtitre plates for 1 h. Secondary antibodies of peroxidase-conjugated goat anti-mouse IgG (Dianova, Hamburg, Germany) were used at 1:5000 dilution. The ELISA absorbance values from normal sera mice were defined negative for each peptide as well as positive values from rMntC-specific sera, for which standard deviations were calculated by three times mean absorbance value.

### ELISA assay for rMntC and immunodominant epitope peptide antibodies

Seven days after last immunisation, the sera of anti-rMntC and anti-immunodominant peptide were collected from the tail vein for analysis of IgG and other antibodies using ELISA. The assay of antibody detection by using ELISA was as described previously[21]. The corresponding collected serum samples from mice immunised with rMntC and epitope vaccination were employed as the primary antibodies and the appropriate horseradish peroxidase-conjugated goat anti-mouse IgG (Dianova, Hamburg, Germany), or goat anti-mouse IgG1, or goat anti-mouse IgG2a, or goat anti-mouse IgG2b (Southern Biotech, Birmingham, AL, USA) were used as the secondary antibody. The highest absorbance was calculated by log test from the reduplicate assay and blank control and the comparison between the two results was undertaken by *t-*test.

### Sequence alignment and localisation

MntC sequences from different *S*. *aureus* strains were retrieved from the GenBank database for alignment by the MEGA software. Immunodominant peptides were mapped against the 3-d structure of MntC (PubMed protein database), as previously reported [22], using the PyMOL 1.1 program with the crystal structure of MntC.

### Opsonophagocytic killing assay

Seven days after last immunisation, the sera of anti-rMntC and anti-immunodominant peptide were collected from tail vein for analysis of opsonophagocytic killing assay (OPKA). The assay was performed using a method previously described by Burton and Nahm [23]. In this study, the assay was performed on the HL-60 cell line (ATCC, CCL-240) cultured in IMDM medium (HyClone) supplemented with 10% foetal bovine serum ((HI-FBS, HyClone). 2 × 10^6^ cells/ml HL-60 cells were differentiated into neutrophil-like cells by the addition of 100 mM dimethylformamide (DMF, Shanghai Shenggong) to the growth medium for 4 days and re-suspended in OPK buffer at a concentration of 2 × 10^7^ cells/ml. At the same time, *S*. *aureus* strains were cultured as before and diluted in OPK buffer to contain 2 × 10^7^ CFU in suspension. Finally, the assay was performed in 2 ml Eppendorf tubes, with each well containing 100 μl HL60 cells, 100 μl bacterial suspension of each *S*. *aureus* strain, 100 μl MntC vaccinated mouse antisera, and 100 μl complement (C′) from infant rabbit serum. After incubation for 2 h at 37°C with shaking, the samples were plated in duplicate, and the killing effect was defined as a reduction in CFU, compared with the negative control normal mouse serum (NMS) after overnight growth. The percentage of opsonophagocytic killing was determined by subtracting the number of colonies surviving the test assay from the number of CFU in the NMS control.

### Statistical analysis

The non-parametric log rank test was performed to determine differences in the survival times. The match T test and Mann-Whitney U test were used to compare antibody levels and opsonophagocytic percentages, respectively. Analyses were performed using GraphPad Prism 5.0 software: *P* < 0.05 was considered statistically significant.

## Results and Discussion

### Expression and purification of rMntC

Recombinant protein was expressed in the soluble fraction in *E*. *coli* under the induction of 0.1 mM IPTG, and the cell lysate was subjected to affinity and ion-exchange chromatography purification by using glutathione-Sepharose and CaptoTM MMC, respectively. As shown in S1A Fig and S1B Fig, we cloned and expressed the complete MntC protein, the recombinant proteins corresponded to their predicted molecular masses (33 kDa) After the primary purification by Glutathione Sepharose 4B beads to remove the GST signal sequences, no visible degradate and aggregate fragment protein was observed under SDS-PAGE. The protein purity reached 99% as determined by HPLC (S1C Fig). The protein concentration was 1.6 mg/ml.

It has been reported that the residual endotoxin and nucleic acid in the protein will largely affect the immunogenicity of the protein [24], and endotoxins, after injection, often induce side effects such as fever. In this study, after extraction of the endotoxin by Triton X-114, the concentration of endotoxin was reduced to less than 2.5 pg per μg protein (data not reported), which was acceptable for injection. As analysed by gel filtration, the absorbance at 280 nm was three times higher than that at 254 nm (S1C Fig), suggesting that the protein obtained contained low amounts of nucleic acid.

### Immunisation with rMntC vaccine can raise protective effect and antibody response against *S*. *aureus* infection

**Fig 1:**
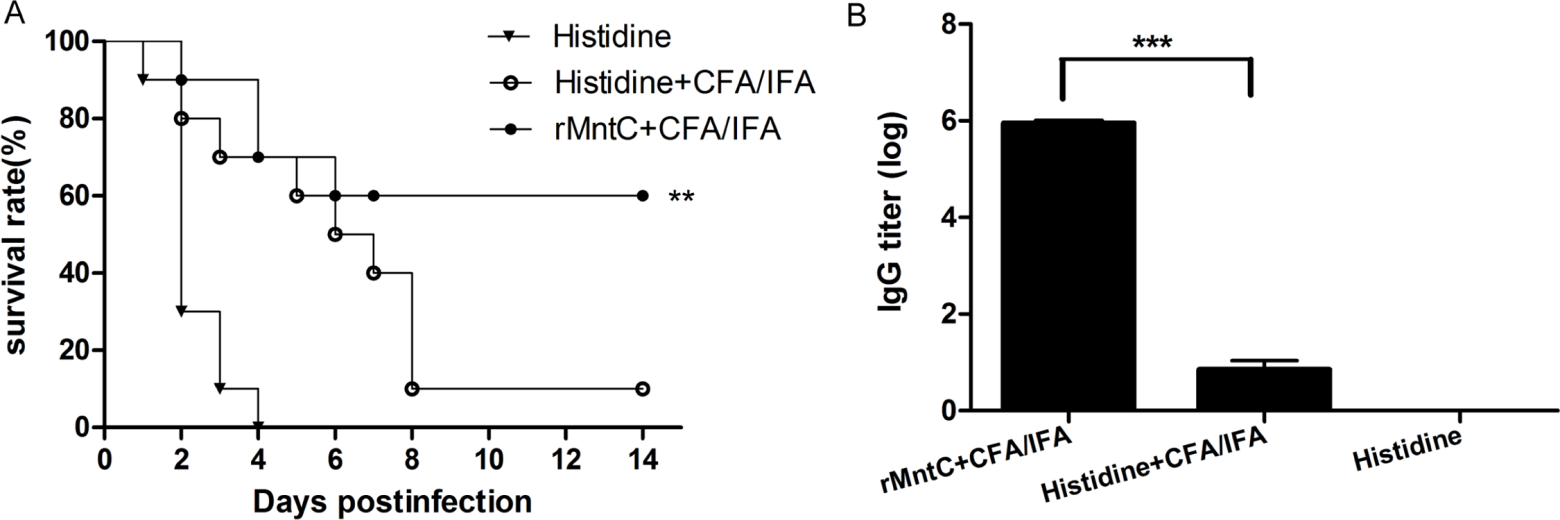
The survival rates and antibody titres of rMntC vaccine. The survival rates of rMntC vaccine (with CFA/IFA as adjuvant), CFA/IFA adjuvant alone, and histidine buffer as control, challenged for BALB/c mice (*n* = 10) by *S*. *aureus* strains MRSA252 (A). The titres were determined by ELISA for IgG (B). The survival analyses and comparison of rMntC vaccine and adjuvant control were calculated separately by a log rank test. The asterisks represent a statistically significant difference (**P < 0.01, ***P < 0.001).

To evaluate the protective effect of rMntC against *S*. *aureus* infection, a systematic infection model was established in BALB/c mice by infection *via* the tail vein using a lethal dose of *S*. *aureus* strain MRSA252. As shown in Fig 1A, the survival rate of mice vaccinated with rMntC was 60%, which was significantly higher than in the CFA/IFA group (*P* < 0.005). In this assay, with the CFA/IFA adjuvant, rMntC antigen from *S*. *aureus* elicited a high protective immunity. Overall, these results suggested that immunisation with rMntC was capable of protecting mice against a lethal challenge with *S*. *aureus*.

Anderson *et al*. [16] proved that Anti-MntC monoclonal antibodies were able to bind *S*. *aureus* and *S*. *epidermidis* cells *in vitro* and also exhibited a protective immunity in an infant rat passive protection model and induced neutrophil respiratory burst activity. As MntC and its *S*. *epidermidis* ortholog SitC are 77% identical in their amino acid sequence [25], the antibody generated by MntC also exhibited protection across the *staphylococcal* species group. The results obtained from the passive protection model suggested that active immunisation with rMntC might also effectively protect against *S*. *aureus* infection. Thus, the protective immunity of rMntC was evaluated in this study, and the results were consistent with passive immunisation. Currently, as a component of a multi-antigen vaccine, MntC has been used in clinical trials that include capsule polysaccharide conjugates and clumping factor A with the potential to prevent *S*. *aureus* infection and disease. These results indicate that MntC could serve as a promising candidate for the development of *S*. *aureus* vaccine, and it may also elicit protective immunity across the *staphylococcal* species group.

The humoral immune response is considered to play an important role in clearing *S*. *aureus* infection, and anti-MntC mAbs are protective in several animal models of infection when used in passive immunisation [16] [26]. Here, we further characterised the humoral immune response to evaluate the efficacy of the rMntC protein in actively immunised mice. One week after the last booster immunisation, serum was collected and MntC-specific IgG antibodies were determined using ELISA.

Using recombinant MntC protein plus CFA/IFA adjuvant, we analysed antigen-specific antibody responses from the pooled sera of immunised mice. As shown in Fig 1B, the titres of rMntC-specific IgG were significantly higher than those of the control group. This result indicated that rMntC exhibited strong immunogenicity and it was able to induce an intense humoral immune response in mice, which may play an important role in protective immunity against *S*. *aureus*.

### Mapping of the three immunodominant epitopes on MntC

In view of the strong humoral immune response of MntC antigen against *S*. *aureus*, the mechanism of antibody immunity was explored. Previous reports indicate that B-cell epitopes, as the least immune unit, are strong enough to elicit a potent humoral immune response without harmful side effects to the human body [27], therefore, the identification of B-cell epitopes of MntC is important in understanding the immunotherapeutic, and immunodetection, characteristics of this as a vaccine antigen. Then we identified the mapping of linear B-cell immunodominant epitopes of the MntC with 18-mer overlapping peptides in this study, which was rationally adopted to identify linear B cell epitopes at low cost and with high efficiency [28].

**Fig 2:**
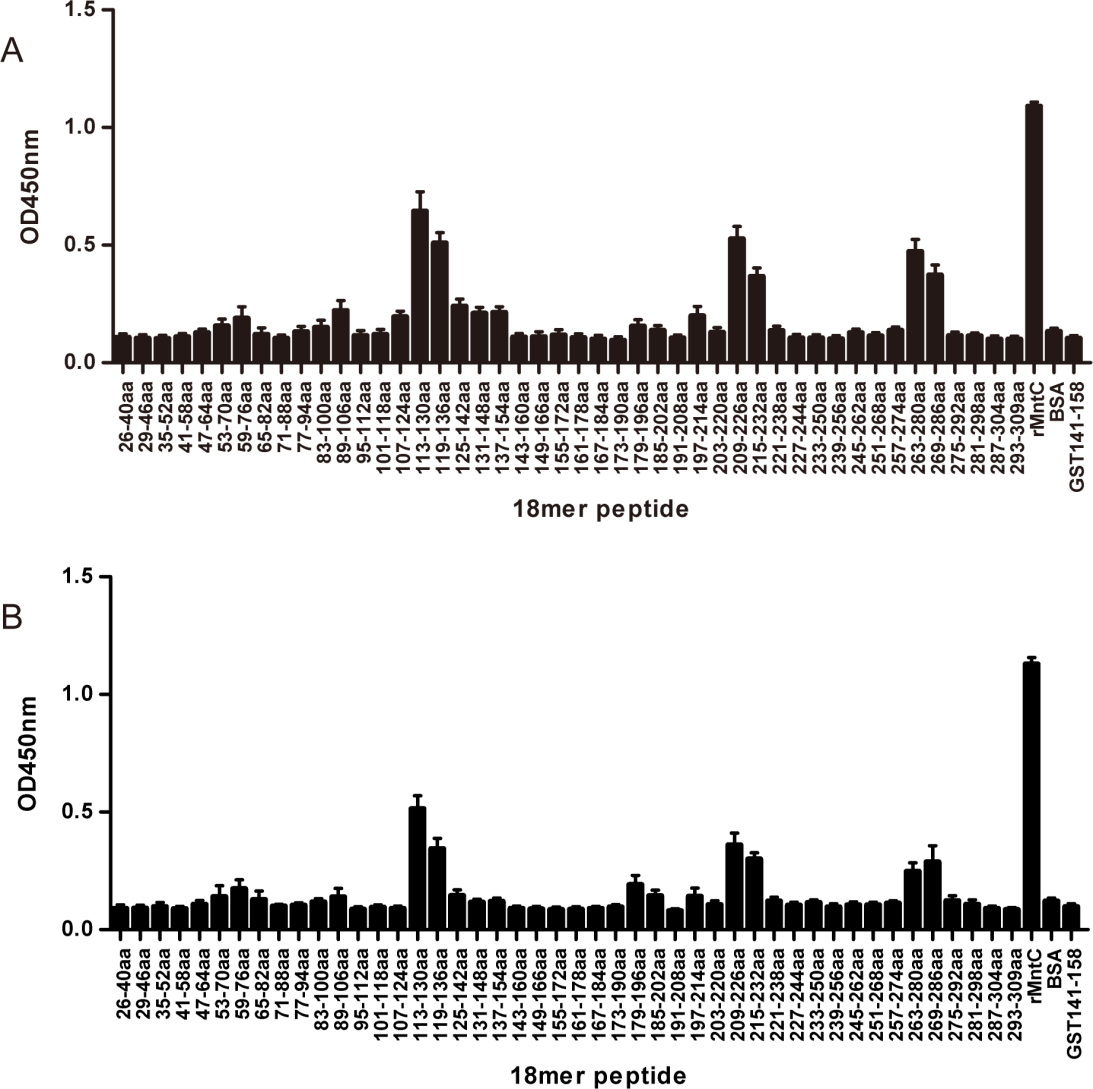
ELISA detection of B cell epitope mapping of MntC with antiserum of immunised and infected BALB/c mice. To determine the immunodominant peptides of MntC, microtitre plates were coated with synthetic overlapping peptides that spanned the entire length of the MntC of MRSA252 or GST141-158 (negative control peptides) or MntC protein and BSA. B cell epitope mapping of MntC using an overlapping 18-mer peptide ELISA with antiserum samples from BALB/c mice that were immunised with rMntC plus CFA adjuvant (A) and antiserum samples from BALB/c mice that were infected after immunisation with rMntC plus CFA adjuvant (B) were detected. The absorbances read at 450 nm for peptides MntC113-136, MntC209-232, and MntC263-286 were significantly higher than BSA (*P* < 0.05) and higher than GST141-158 (*P* < 0.01).

Crossing the entire length of the MntC of MRSA252, we synthesised forty six 18-mer overlapping peptides to identify the linear B-cell epitope mapping of MntC by ELISA, and the antiserum samples form rMntC-immunised mice and MRSA-infected mice post rMntC vaccinations were tested respectively. As shown in Fig 2, the strongest IgG antibody reactivity results of immunised mice antiserum(Fig 2A) were consistent with infected mice antiserum with immunised before infection (Fig 2B), which concentrated on the three major immunodominant peptides: MntC113-136 (KKVIAVSKDVKPIYLNGEEGNKDK), MntC209-232 (FKYFSKQYGITPGYIWEINTEKQG), and MntC263-286 (MESLSEETKKDIFGEVYTDSIGKE) of MntC antigen and indicated a homologous immunodominant antibody response. Therefore, peptides of MntC113-136, MntC209-232, and MntC263-286 may contain novel linear B-cell immunodominant epitopes that induced major immunodominant huromal responses in MntC vaccination. This is the first study to map the B-cell epitopes of MntC to date, which nearly contained all of the linear B-cell epitopes of MntC from MRSA252. It may be helpful for understanding the immune protective efficacy of MntC antigen against *S*. *aureus* infection.

### Localisation of immunodominant epitopes on MntC

**Fig 3:**
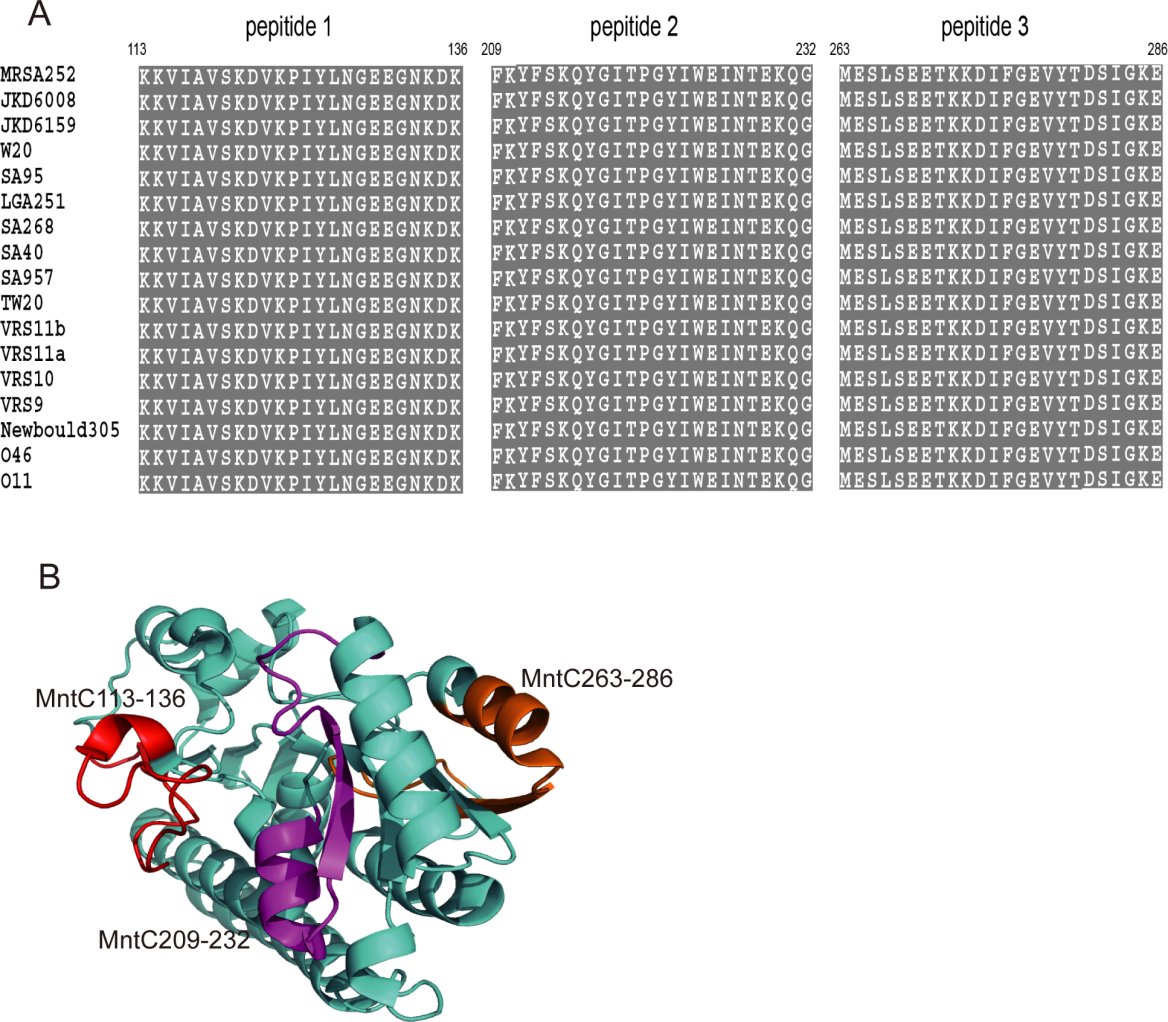
Sequence alignment and localisation of immunodominant epitopes on MntC. Sequence alignment of immunodominant peptides of MntC demonstrated that the amino acids of MntC113-136, MntC209-232, and MntC263-286 of MntC were wholly conserved in all *S*. *aureus* strains (A). Localisation of immunodominant peptides MntC113-136, MntC209-232, and MntC263-286 on the 3-d crystal structure of MntC. MntC113-136, MntC209-232, and MntC263-286 are shown in red, magenta, and orange respectively (B).

The amino acid sequences of MntC113-136, MntC209-232, and MntC263-286 of MntC from the selected MRSA252 strain and other *S*. *aureus* strains were retrieved from the GenBank database to analyse the conservation of immunodominant epitopes. Sequence alignments indicated these three peptides were completely conserved among different strains (Fig 3A). Since the 3-d crystal structure of MntC was available in the PDB database (PDB code: 4K3V), we located the three peptides in the structure in MntC by using the PyMOL 1.1 program. As shown in Fig 3B, MntC113-136 (red), MntC209-232 (magenta), and MntC263-286 (orange) were located on the surface of MntC and were accessible to host immune cells and immunoglobulin. The homology analysis revealed that MntC113-136, MntC209-232, and MntC263-286 were well-conserved among different *S*. *aureus* strains, which indicated that these immunodominant epitopes might be novel candidate agents for vaccines based on MntC.

Moreover, a previous study [22] reported that the MntC molecule consists of a 3-d structure from *S*. *aureus*, including the N-terminal (residues 14–148aa), C-terminal (179–291aa) domains, and the domain-connecting α-helix (149–178aa). Based on the 3-d crystal structure mapping results, these three immunodominant epitopes contained the Mn binding coordination. Furthermore, due to location on the surface of MntC, it was easier to induce a stronger antibody response for these epitopes, which can explain the intense humoral immune protection. Further studies of potential polypeptide vaccines based on the three immunodominant epitopes are recommended.

### Immunisation with polypeptide vaccine can be effective against *S*. *aureus* infection

**Fig 4:**
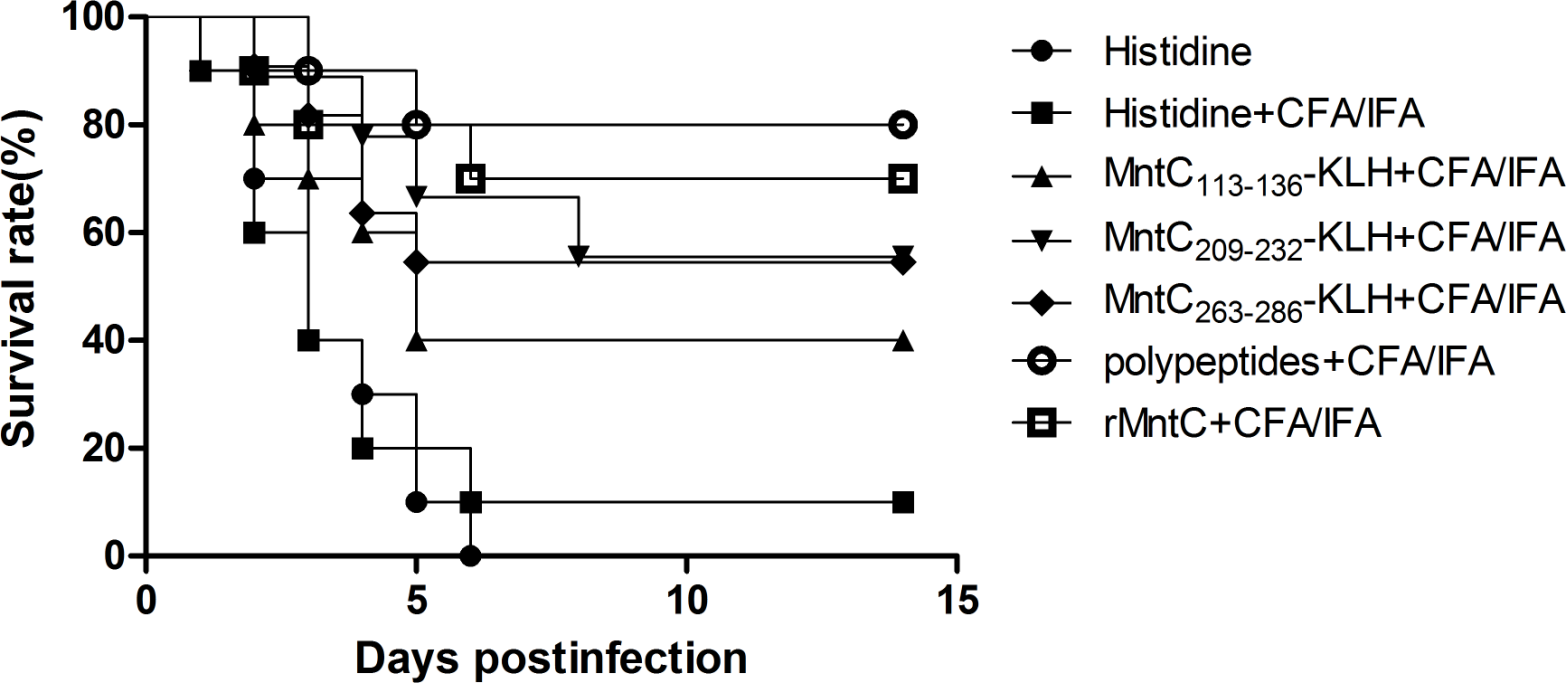
The survival rates of rMntC and peptides vaccine. The survival rates of rMntC and conjunct peptides with KLH vaccine (with CFA/IFA as adjuvant), and CFA/IFA adjuvant alone as control, challenged for BALB/c mice (*n* = 10) by *S*. *aureus* strains MRSA252. The immunized dose of peptides vaccine was 40μl(100μg peptides-KLH) with 40μl CFA/IFA adjuvant and the mixture polypeptides vaccine was prepared with equal distribution of all three immunodominant epitopes (33.3μg/peptide-KLH) with 40μl CFA/IFA adjuvant. The survival rate comparison of rMntC and polypeptide vaccine were insignificant, but survival analyses and comparison of polypeptide vaccine and adjuvant control were significant when assessed by log rank test (*P* < 0.01).

In view of the protection of MntC antigen in the *S*. *aureus* infection immunity, MntC113-136, MntC209-232, and MntC263-286 of MntC, which are the conserved immunodominant epitopes located on the surface of MntC, would also be able to prevent infection from *S*. *aureus* as an epitope-peptide vaccine. To evaluate the immune protective effect of this epitope-peptide vaccine against *S*. *aureus* infection, adjuvant with identical volume CFA/IFA, the 100μg peptides of MntC113-136-KLH, MntC209-232-KLH, and MntC263-286-KLH respectively were designed as individual vaccines, and the mixture polypeptides vaccine, which is comprised of equal distribution of all three immunodominant epitopes (33.3μg/peptide-KLH) and rMntC antigen vaccine were also immunised in an *S*. *aureus* strain MRSA252 systematic infection BALB/c mice model. Results indicated that polypeptide and rMntC antigen vaccines exerted excellent immune protective effect, which was significant compared to the monovalent peptide vaccine and adjuvant control. Details in Fig 4 showed that the survival rate of polypeptides and rMntC vaccine were 80% and 70%, respectively, which were significantly higher than MntC113-136-KLH, MntC209-232-KLH, and MntC263-286-KLH peptide vaccines; it suggested that immunisation with a polypeptide vaccine could generate intense protection against lethal challenge with *S*. *aureus* MRSA252. As an effective *S*. *aureus* vaccine antigen component, rMntC was demonstrated to be a potentially valuable therapeutic target for the development of antibiotics [29] and the immunodominant of B-cell epitope showed an identical, or even better, function at low cost and high efficiency in protecting against *S*. *aureus* infection.

### Immunisation with polypeptide vaccine induced strong antibody response

**Fig 5:**
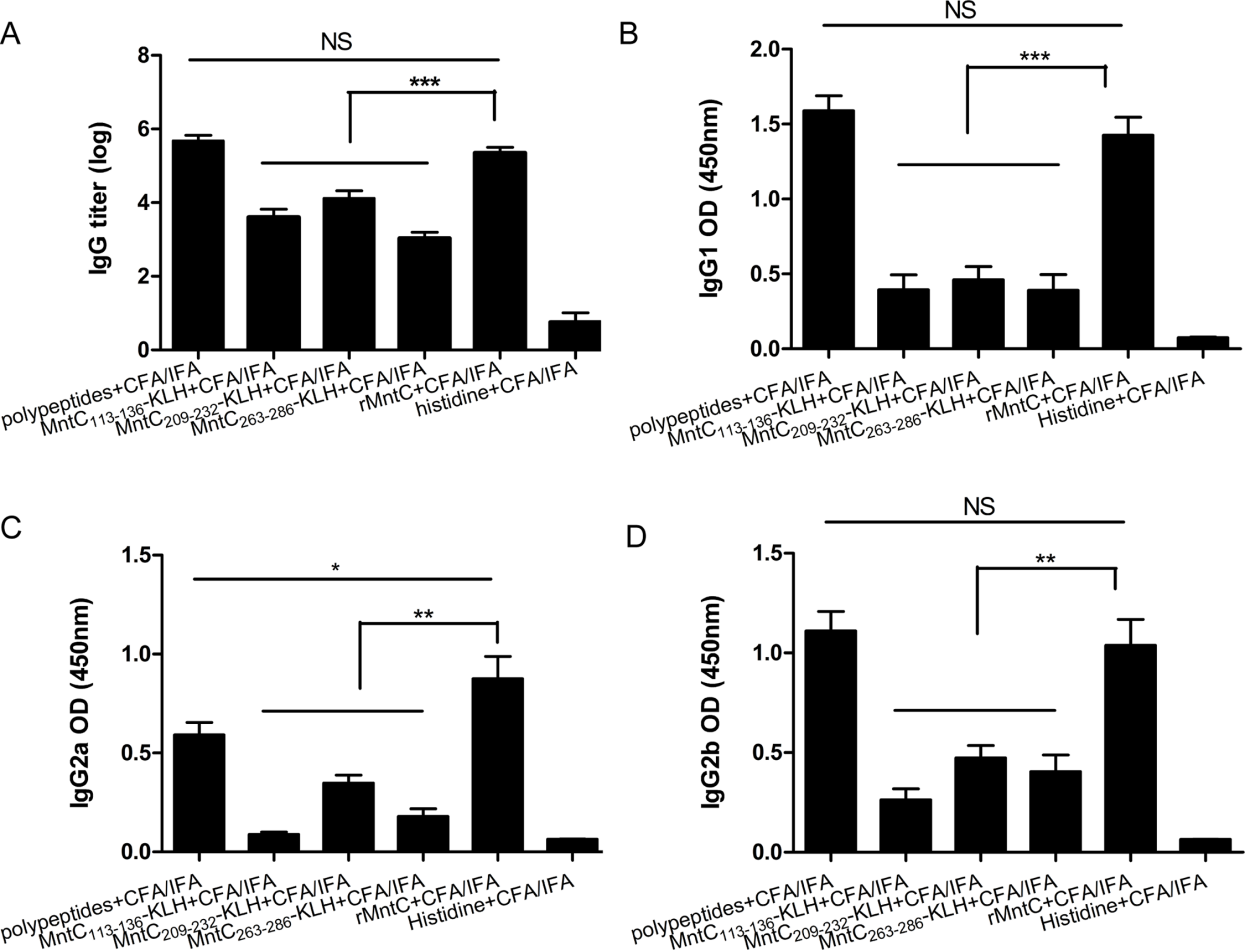
Antibody titres of immunisation with rMntC, peptide vaccine, and controls. The titres were determined by ELISA for IgG (A), IgG1 (B), IgG2a (C), and IgG2b (D). The results represent the means and standard error for a group of mice and the significance was measured by a log rank test (*P < 0.05, **P < 0.01, ***P < 0.001).

The titres of specific antibodies in the sera against the different peptide, and rMntC protein, vaccines were tested by ELISA. The polypeptide, and rMntC plus CFA/IFA adjuvant, produced intense antigen-specific antibody responses. As shown in Fig 5A, levels of antigen-specific IgG were increased in each vaccinated group, and in particular in the antibody response of mice immunised with polypeptide and rMntC vaccines, which exhibited significantly higher serum IgG levels than monopeptide and adjuvant control (*P* < 0.001).

It is well-known that markers of IgG2a and IgG1 represent Th1 and Th2 responses, respectively. In Fig 5B and 5C, the levels of antigen specific IgG2a and IgG1 showed that the IgG1-to-IgG2a ratio ranged from 0.45 to 3.3, which suggested that a Th2-biased response had been induced. These indicators inflected humoral immune response to take advantage of immune protection in the polypeptide vaccine. Accordingly, the IgG2a expressions of immunised polypeptides were inferior to rMntC vaccine, which indicated that antibody responses generated bias to peptides, especially in the polypeptide vaccine group. In addition, levels of IgG2b antibody suggested that the humoral immunity response, as well as serum IgG2a titres, were analysed as being the TH1 cellular immunity response. In summary, it was notable that immunisation with polypeptides was efficacious and could induce prominent IgG antibody responses relative to individual peptide groups or the rMntC antigen group.

Earlier studies showed that vaccine-specific antibody responses may contribute to protection, and there is evidence suggesting that vaccine-induced Th2 responses mediate protection [30]. In addition, the mechanism of the specific MntC-antibody was reported as inhibiting bacterial MntC with an antibody fragment targeting its periplasmic substrate binding protein (SBP) and the antibody prevents the interaction of SBP with the membrane transporter, blocking substrate import [31]. The importance of antibody to MntC suggested a functional correlation between antibody titres for immunodominant polypeptides as well as protective immunity.

### Antibodies of polypeptide vaccines had better opsonophagocytic killing activity *in vitro*

**Fig 6:**
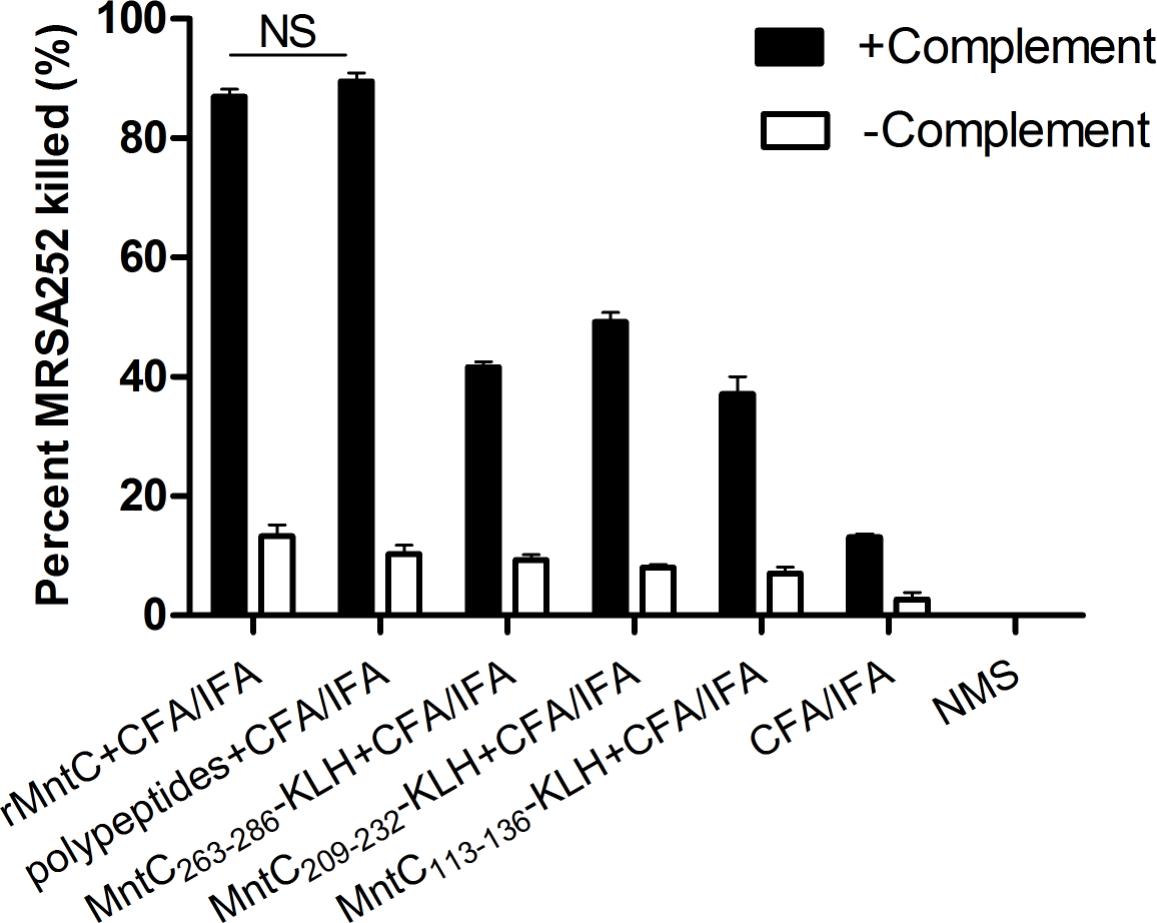
OPKA of antisera to peptides (Anti-peptides) or rMntC (anti-rMntC) against MRSA252. 2 × 10^6^ CFU *S*. *aureus* strains were incubated in the presence of 2 × 10^6^ HL60 cells and mouse antisera against rMntC and peptide vaccine in the presence of infant rabbit complement. A percentage of MRSA252 was killed in the opsonophagocytic assay. Bars represent percentage killing compared with NMS represented as a mean of quadruplicate samples with SEM. NMS: no mouse antisera. No complement: no infant rabbit complement.

Although immunisation with polypeptides can induce a strong humoral immune response *in vivo*, whether these peptide-specific antibodies provided protective immunity *in vitro* remained unclear. To evaluate the efficacy of the peptide-specific antibody, an opsonophagocytic killing assay (OPKA), which measured antibody and complement-mediated bacterial killing, was performed *in vitro*. The killing assay was conducted by using mouse antiserum of specimens immunised with peptide, and rMntC, vaccinations and neutrophil-like cells HL60, which play an important role in host clearance of *S*. *aureus*. In the presence of HL60 phagocytic cells and infant rabbit serum complement, mouse IgG raised by various vaccines exhibited different opsonophagocytic killing activities for *S*. *aureus* MRSA252 strains. As shown in Fig 6, the percentages of *S*. *aureus* killed by the peptide-MntC113-136, peptide-MntC209-232, and peptide-MntC263-286 specific antibody were smaller than the desired percentage (usually 50%) [23], which was only slightly significant. However, opsonophagocytic killing activities of rMntC-specific and polypeptide-specific antibody were 86.9% and 89.5%, respectively: these were still significant even when using 1:3 diluted antibody serums (*i*.*e*. greater than 50%) (Results are not shown). The polyclonal antibodies of polypeptide vaccine were effective in preventing bacteraemia, and were superior to other vaccine groups’ trialled in this study. This indicated that the protective effect of the polypeptide vaccination was mostly antibody-mediated. Opsonophagocytic properties of antibodies were assayed *in vitro*, and showed that specific-polypeptide antibodies behaved more effectively in phagocytosis of the bacteria. These results confirmed that polypeptide-specific antibody was able to kill *S*. *aureus* cells efficiently *in vitro* and may be responsible for the prevention of full-blown infection and bacterial persistence.

## Conclusions

Epitope-based vaccines, constituting of immunodominant epitopes from major protective antigens, are likely to induce strong, comprehensive protective immunity as well as prevent undesired side-effects. Studies[32] [33] of B cell and T cell epitope vaccines were discussed from the perspective of providing protecting against several infectious diseases, these provide references for further development of vaccinations. At present, HIV [34] [35], Neisseria meningitides [36] and Mycobacterium tuberculosis [37] epitope vaccines have been developed to protect against infections. Epitope-based vaccines may also be a potential and effective preventive strategy in *S*. *aureus* infection.

Some important protective antigens against *S*. *aureus* have been identified as vaccine candidate antigens such as SEB [38] and MntC [16]. Although the MntC protein as a valid antigenic molecule, was reported as efficacious against *S*. *aureus* [39] infection and the protein was discussed from the perspective of about function and mechanism in previous studies [40] [41], the immunodominant epitopes, and vaccines based thereon, have not been investigated researched until now. In this study, we reported that potential B cell epitopes from MntC were screened to construct a polyvalent epitope vaccine, and CFA/IFA were used as adjuvants because of their induction of the T cell repertoire to a more TH2 phenotype [42].

In this study, we verified the immune protective efficacy of the rMntC antigen of *S*. *aureus*, and identified the key effective B-cell immunodominant epitopes of its humoral response. Peptides of MntC113-136, MntC209-232, and MntC263-286, as the immunodominant epitopes, were analysed by sequence alignments and were located on the surface of the 3-d crystal structure of MntC. Furthermore, we provided preclinical evidence for a polypeptide vaccine based on the three immunodominat epitopes of MntC against *S*. *aureus* as a component of a potential multi-antigen prophylactic anti-staphylococcal vaccine. Immunisation with the polypeptide vaccine, which contained three immunodominant epitopes, was able to confer protective effect against *S*. *aureus* disease in *S*. *aureus* bacteraemia infection, as assessed by an evaluation of survival rates, antibody response, and opsonophagocytic activity. In conclusion, these results indicated that three novel immunodominant epitopes–MntC113-136, MntC209-232, and MntC263-286 –played a vital role in the protection of MntC and could be a rational, cost-effective, and simple component in the development of a design for an epitope vaccine against *S*. *aureus* infections.

## Supporting Information

S1 Fig Expression, purification, and characterisation of rMntC as a vaccine candidate antigen for MRSA. Schematic diagram showing the primary structure of rMntC (A); Purified recombinant protein (rMntC) analysed by SDS-PAGE (B) and gel filtration(C).
(TIF)
