# Supplementary figures and images for: Immunisation With Immunodominant Linear B Cell Epitopes Vaccine of Manganese Transport Protein C Confers Protection against Staphylococcus aureus Infection
Source: PLoS One. 2016 Feb 19;11(2):e0149638. doi: 10.1371/journal.pone.0149638 (PMC4764517; doi:10.1371/journal.pone.0149638)

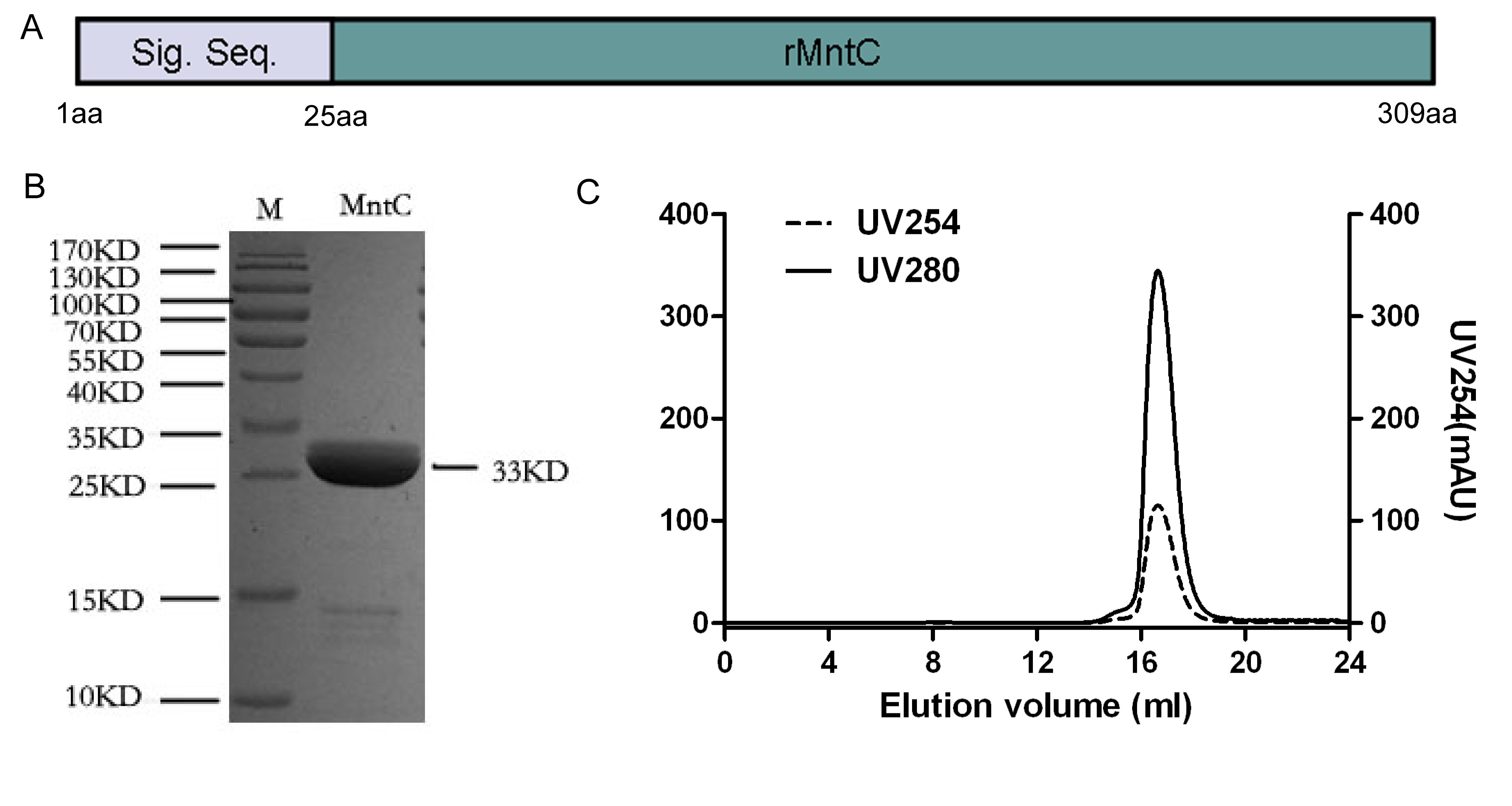

Supplement: S1 Fig — Schematic diagram showing the primary structure of rMntC (A); Purified recombinant protein (rMntC) analysed by SDS-PAGE (B) and gel filtration(C). (TIF) [file pone.0149638.s001.tif]
